# Evaluation of Food Insecurity in Adults and Children With Cystic Fibrosis: Community Case Study

**DOI:** 10.3389/fpubh.2018.00348

**Published:** 2018-11-26

**Authors:** Perry S. Brown, Dixie Durham, Rick D. Tivis, Shannon Stamper, Cleary Waldren, Sarah E. Toevs, Barbara Gordon, Tiffany A. Robb

**Affiliations:** ^1^St. Luke's Cystic Fibrosis Center of Idaho, Boise, ID, United States; ^2^Sam and Aline Skaggs Health Science Center, Idaho State University, Meridian, ID, United States; ^3^St. Luke's Health System, Boise, ID, United States; ^4^Center for the Study of Aging, Boise State University, Boise, ID, United States; ^5^Community and Environmental Health, Boise State University, Boise, ID, United States

**Keywords:** cystic fibrosis, food insecurity, body mass index, pulmonary function, advocacy, research, assistance programs

## Abstract

Advances in the care and treatment of cystic fibrosis (CF) have led to improved mortality rates; therefore, considerably more individuals with CF are living into adulthood. With an increased number of CF patients advancing into adulthood, there is the need for more research that surrounds the aging adult CF patient. It is important to conduct research and collect results on the aging CF population to help better prepare the CF patient, who is dealing with the heavy treatment and financial burden of their disease, build autonomy and increase their quality of life. Of note, research has found that social, behavioral, and physical factors influence the ability of those with CF to follow dietary recommendations. A primary treatment goal in CF is a high calorie, high protein, and high fat diet. A socio-economic factor that has not been adequately investigated with regards to dietary compliance of individuals with CF is food insecurity. The aim of this community case study was to document the experiences and estimate the prevalence of food insecurity among CF patients residing in Idaho. The correlation between food insecurity and health outcomes (lung function and body mass index) was also examined. Participants included adult patients and parents of pediatric patients with CF. Food insecurity rates among CF patients of all ages were found to be significantly higher than that seen in the overall community; however, no specific correlation between food insecurity and body mass index (BMI) or lung function emerged. This case study highlights the need for continued research around food access issues in this patient population. The data resulting from this study shows the value of CF advocacy organizations promoting efforts to build resources and provide education around food insecurity issues.

## Introduction

Recent advances in the care and treatment of CF have led to improved mortality rates; therefore, considerably more individuals with CF are living into adulthood (1, 2). In 2014, 50.7% of individuals with CF in the United States were over the age of 18 (3). Furthermore, studies report median survival rates of more than 50 years for individuals with CF born in the year 2000 or later (4). Thus, there is a need to better understand the impact of long-term care and quality of life issues among the aging CF population.

In addition to a range of medical treatments, dietary modifications are required to manage CF. Hypercaloric diets (high-fat, high-protein) are prescribed to reduce malnutrition risk and obtain/maintain optimal growth (5–8). Nutritional supplementation (orally or enterally) may also be required to promote adequate intake of energy and nutrients (6, 8). Of note, compliance with the CF diet is generally poor; estimated adherence is 40–55% (9, 10). Research has found that social, behavioral, and physical factors influence the ability of individuals with CF to follow dietary recommendations (5, 8, 11, 12). Though Sullivan and Mascarenhas advocate for food insecurity screening by health professionals working with the CF population (13), food access as a socio-economic factor has not been adequately investigated as a nutritional risk factor among individuals with CF. This community case study aimed to document the experiences and estimate the prevalence of food insecurity in an Idaho-based CF population. The correlation between food insecurity and health outcomes was also assessed.

## Background and rationale

CF is a genetic condition causing physiological impairments resulting in the production of thick, sticky secretions in several glands and organs. A buildup of mucus in the lungs leads to persistent and progressive lung infections; thereby, a patient's ability to breathe independently diminishes, and the risk for respiratory failure escalates. A buildup of mucus in the pancreas impairs digestion and increases a patient's risk for a myriad of vitamin and mineral deficiencies as well as malnutrition (14). A large, multi-site trial reported a malnutrition rate of 26.8% among people with CF in the United States (15).

In 2013, CF disease management costs for patients in the United States were estimated at $15,571 per patient per year; $306,000 per patient across a lifetime; however, based on clinical experience, the financial burden may be more substantial (16). Given that CF is a progressive condition, as a patient ages, medical costs also escalate (16, 17). In the United States, annual costs for individuals with mild, moderate, and severe symptoms are estimated at $10,151, $25,647, and $33,691, respectively (16).

Patient self-care or parent/caregiver tasks can also require an extensive time commitment and cause an impact to financial status. For example, the average patient/parent must spend 2 h every day performing complex homecare therapies, one of which is ensuring adequate dietary intake (1, 18). If a CF patient develops an acute pulmonary exacerbation, additional treatments are required and hospitalization for an extended period (two weeks or longer) may be necessary (18). In addition to high medical expenses, complications of CF may result in lost work time for patients and/or family caregivers translating to overall lower household incomes and financial strain (16).

The United States Department of Agriculture (USDA) defines food insecurity as a household-level economic and social condition of limited or uncertain access to adequate food (19). In 2014, the food insecurity rate in the US was 13.7% (19). Idaho estimates were 14.7%, or about one in seven Idahoans (19–21).

Nearly one-third (30.6%) of individuals with chronic disease experience food insecurity (22). Prior to this study conducted at the St. Luke's CF Center of Idaho, the incidence of food insecurity among the CF population living in Idaho had not been well-studied. However, McDonald et al. (23). evaluated nutrition knowledge and food security among parents of pediatric CF patients attending clinics in the Mountain West region—Arizona, Colorado, New Mexico, and Utah. The overall level of food insecurity among families with at least one child with CF was 26.3%, double the average rate of food insecurity for the general population residing in that geographic region (23). Furthermore, there appears to be a dearth of research on the prevalence of food insecurity among adult CF patients, which proves this case study innovative in looking into the aging adult CF population.

Failure to comply with CF dietary guidelines because of food insecurity can put an individual with CF at greater risk for impaired nutritional status and, ultimately, malnutrition. Malnutrition, in turn, may lead to a spiral of increased pulmonary infections, diminished lung function, and ultimately decreased survival (8, 24). In contrast, optimized or improved nutritional intakes have been shown to postpone declines in pulmonary function, thereby decreasing early mortality (8, 25). Food insecurity is also associated with higher rates of anxiety and depression—and these mental health conditions may decrease a CF patient's adherence to therapies needed to maintain optimal health (12, 26).

## Setting

This community study was implemented at the St. Luke's CF Center of Idaho between August 2013 and March 2015. The protocol, which included a computer-based survey plus the collection of both lung and body mass biometrics, was conducted during routine quarterly visits with a multidisciplinary team of health care providers. The St Luke's Health System Institutional Review Board evaluated and approved all aspects of the study. Consent was obtained using the St. Luke's Institutional Review Board-approved form.

Data collection tools were developed using Research Electronic Data Capture (REDCap) which is HIPAA-compliant and designed to support collection and management of confidential data for clinical research studies and made available from the Institute of Translational Health Science (ITHS) via grant UL1 RR025014 (27).

The survey instrument employed for this study was the Mountain West Cystic Fibrosis Consortium Questionnaire (MWCFC-Q). MWCFC-Q was developed by McDonald et al., who used it to assess food insecurity among CF pediatric patients residing in the western United States (23). Due to wanting to investigate both pediatric and adult CF patients, the tool adapted questions from both the Childhood Hunger Identification Project and the Short Form of the 12-month food security scale of the USDA, Food and Nutrition Service (28). Content and face validity, as well as reading level (grade 7–8), were established for the tool. In addition to queries regarding access to food, information on usage of food assistance programs was collected. Demographics (age, gender, and educational levels for both patients and parents) were also requested.

Lung function metrics were collected for participants over the age of 6 years utilizing a MicroLoop spirometer; forced expiratory volume in 1 s (FEV_1_) was measured using National Health and Nutrition Examination III (NHANES III) predicted equations (29, 30). FEV_1_ is considered to be a reliable measure of therapeutic efficacy and disease progression; an FEV_1_ >80% predicted value is associated with greater lung function and better nutritional status (8, 12). Height, weight, BMI, and compliance with dietary modifications were assessed by a registered dietitian nutritionist (RDN). In addition to an indicator of nutritional status, among the CF population, BMI strongly correlates with lung function and rate of lung function decline (8, 12, 13). For individuals with CF, clinical guidelines for ideal BMI correlate to achievement of optimal lung functioning (FEV1); depending upon the patient's age, clinicians employ either the World Health Organization (WHO) or Centers for Disease Control and Prevention (CDC) reference charts (13). For children under 2 years, a BMI of > 50 percentile per WHO charts is optimal; for those 2 years and older, the ideal BMI is ≥50 percentile per the CDC standards. CDC BMI goals are used to assess BMI among adults with CF, goals are ≥ 22 kg/m^2^ for women and ≥23 kg/m^2^ for men (13, 31).

Data were collected from patients/parents using a tablet computer. The research coordinator entered BMI and pulmonary function data into the patient survey that was accessible on the tablet. Then, the password protected tablet was provided to the adult CF patients or the parents of children with CF for completion of the survey questions.

Statistical analyses were performed using Statistical Package for the Social Sciences version 23.0, 2015 (SPSS/PC; IBM Corp., Armonk, New York) with a statistically significant *p*-value set at 0.05. Confidence intervals were calculated using the online Sample Size Calculator provided by the University of California San Francisco's National Center for Advancing Translational Sciences.

## Results

Across 20 months, 44 adults with CF and 43 parents of children with CF participated in the study. Most of the CF parents were between 26 and 40 years (72%); 43% of the adult CF patients were within this age range. An equal number of adult CF patients (43%) were between 18 and 25 years. Comparable numbers (37 and 39%) of CF parents and adult CF patients had some years of college education; however, more of the CF parents were college graduates (30 vs. 18%). A small percentage (16%) of parents had more than one child with CF. Table 1 provides a summary of the demographics and biometrics of patients/parents participating in the survey.

**Table 1:** Demographics of study patients with cystic fibrosis.

	**Adult with CF (18-50+ years)**	**Child with CF (< 1 to 17 years)**	Parent of a child with CF^*^
Number of participants	44	43	43
**GENDER**			
Male	21	Not collected	7
Female	23		36
**EDUCATION**			
Some high school	3	Not collected	2
High school	12		8
Some college	17	NA	16
College graduate	8	NA	13
Some graduate school	1	NA	1
Master's degree or higher	3	NA	3
**SUPPLEMENTAL FOOD PROGRAMS**			
Number of participants receiving food assistance	12	29	29
**BODY MASS INDEX**			
Malnourished: < 10th percentile	NA	2	NA
Underweight: < 50th percentile	NA	20
Normal/healthy weight: > 50th to < 95th percentile	NA	9	NA
Overweight/obese: ≥ 95th percentile	NA	2	NA
Underweight: < 18.5 kg/m^2^	4	NA	NA
Normal/healthy weight: 18.5–24.9 kg/m^2^	31	NA	NA
Overweight: 25–29.9 kg/m^2^	7	NA	NA
Obese: > 30 kg/m^2^	2	NA	NA
**FORCED EXPIRATORY VOLUME IN ONE SECOND (FEV**^1^**)**			
Normal: FEV_1_ > 90	6	10	NA
Mild obstruction: FEV_1_ 70–90	10	10	NA
Moderate obstruction: FEV_1_ 40–70	23	4	NA
Severe obstruction: FEV_1_ < 40	4	0	NA
Testing not done (< 6 years old)	NA	19	NA

* *Applicable data was collected on parents who completed surveys for their children. NA, Not applicable*.

The children with CF ranged from 1 to 17 years; adults 18 to over 51 years. Two children had BMIs within the overweight/obese range (>95th percentile); 22 had BMIs indicative of nutritional risk—20 < 50th percentile and 2 < 10th percentile. Two adults had BMIs within the obese range (>30 kg/m^2^), and seven were overweight (25–29.9 kg/m^2^); four had BMIs indicative of nutritional risk (< 18.5 kg/m^2^). The mean BMI for both adults and children with CF was within normal limits and optimal range. Table 1 contains a breakdown of BMI for the CF patients in this study.

The average FEV_1_ percent predicted value for the children with CF was 84.0 (std. dev. 20.1). The minimum FEV1 percent predicted was 42 and the maximum was 124. For adults with CF, FEV1 percent predicted ranged from 27 to 110, with an average reading of 64.8 (std. dev. 21.1). More than one third of adults with CF (36.4%) had FEV_1_ percent predicted in the mild disease to normal range (70 to >90%)−46.5% of children. Less than 1% of the adults (4/44) and none of the children in the study had FEV_1_ percent predicted readings in the severe range (< 40%). Overall, the mean FEV_1_ for this patient pool was minimally below normal limits. See Table 1 for more detailed information on pulmonary function readings.

Tables 2, 3 summarize compiled findings of the food insecurity survey. One–third (32.6%; 95% CI 19–49%) of parents of children with CF and 43% (95% CI 28–59%) of adults with CF reported that they worried food would run out before they could afford to buy more groceries. Nearly half of the parents (46.5%, 95% CI 31.2–62.3%) revealed that, in the past 12 months, groceries did not always last until there was a new source of funds to replenish the supply. Adults with CF responded similarly—40.9% (95% CI 26.3–56.8%) said that they always/most of the time/sometimes ran out of food in the last 12 months.

**Table 2:** Results of food security tool completed by parents of children with cystic fibrosis.

	**Always**	**Most of the time**	**Sometimes**	**Never**	**Prefer not to answer**
In the last 12 months, have you worried whether your food would run out before you got money to buy more?	2	0	12	29	0
In the last 12 months, has the food you bought lasted until you had money to buy more?	23	11	7	2	0
In the last 12 months, have you or other adults in the household cut the size of your meals or skipped meals because there wasn't enough money for food?	0	1	6	36	0
In the last 12 months, how often did you serve punch or water with a meal because you didn't have money to buy milk?	0	1	3	39	0
In the last 12 months, have you or your children eaten with friends or relatives because you didn't have money to buy food?	0	0	6	37	0
In the past 12 months, have you bought all the basic foods (meat, milk, cheese, fruits and vegetables) your family eats without worrying about the cost of the food?	20	14	7	2	0
In the last 12 months, have you bought extra foods (butter, sour cream, peanut butter) for your child with CF without worrying about the cost of the extras?	20	14	6	3	0
In the past 12 months has your child with CF used liquid food supplements that are taken by mouth?	16	7	9	10	1
In the past 12 months, have you had to decide between buying medications and food for your CF child?	0	0	3	40	0
In the past 12 months, has your child with CF used any type of feeding given by a tube into the stomach?	6	1	0	34	2

**Table 3:** Results of food security tool completed by adults with cystic fibrosis.

	**Always**	**Most of the time**	**Sometimes**	**Never**	**Prefer not to answer**
In the last 12 months, have you worried whether your food would run out before you got money to buy more?	2	1	16	25	0
In the last 12 months, has the food you bought lasted until you had money to buy more?	26	8	8	2	0
In the last 12 months, have you or other adults in the household cut the size of your meals or skipped meals because there wasn't enough money for food?	1	2	6	34	1
In the last 12 months, how often did you serve punch or water with a meal because you didn't have money to buy milk?	1	3	4	36	0
In the last 12 months, have you eaten with friends or relatives because you didn't have money to buy food?	0	0	11	33	0
In the past 12 months, have you bought all the basic foods (meat, milk, cheese, fruits and vegetables) you or your family eats without worrying about the cost of the food?	16	11	11	6	0
In the past 12 months, have you bought extra foods (butter, sour cream, peanut butter) without worrying about the cost of the extras?	17	9	9	9	0
In the past 12 months have you used liquid food supplements that are taken by mouth?	3	8	14	19	0
In the past 12 months, have you had to decide between buying medications and food?	1	3	7	33	0
In the past 12 months, have you used any type of feeding given by a tube into the stomach?	0	3	2	39	0

About 7% of parents of children with CF indicated sometimes choosing between buying medications and food in the last 12 months (95% CI 1.5–19.1%). The incidence was higher among adults with CF—25% responded that they always/most of the time/sometimes had to make this choice (95% CI 13.2–40.3%).

Children with CF appear to be less food insecure than their adult counterparts. They were more likely to have access to sufficient amounts of food (46.5% of pediatric vs. 36.4% of adult patients) and families of children were more apt to purchase extra foodstuffs (46.5% of families with CF vs. 38.6% adults with CF).

In both adult and pediatric CF populations, no significant correlation emerged between either BMI or FEV_1_ and responses to the two key food insecurity questions: “Were you worried your food would last before you got money to buy more?” and “Has the food you bought lasted until you had money to buy more?” (*p* > 0.05). There was also no significant association found between BMI/BMI percentile and FEV_1_ (*p* > 0.05; *r* = 0.24), or only BMI percentile and FEV_1_ (*p* > 0.05; *r* = 0.16).

Data were also collected on the quality, variety, and desirability of dietary intakes. For instance, 18.2% of adults with CF (95% CI 8.25–32.7%) and 16.3% of parents of children with CF (95% CI 6.8–30.7%) reported that they most of the time/sometimes cut the size of meals or skipped meals because of financial restrictions. Indeed, when asked if they could have any food they wanted, many participants listed protein foods (chicken, hamburgers, seafood, steak, cheese) and potatoes (chips, baked, mashed). These foodstuffs are examples of high calorie, high-fat options recommended in CF dietary guidelines.

Among this population, 76.2% of the children with CF consumed liquid food supplements; in comparison, only 25% of the adult patients in the study did. In addition, 17.1% of the children and 11.4% of the adults employed tube feeding to meet caloric needs. Thus, treatment protocols for these patients include the added expense of purchasing liquid food supplements and tube feed supplies.

The question of the need to utilize food assistance programs due to limited resources was also investigated. Among study participants, 15% of CF adults and 26% of CF parent respondents reported participating in the Supplemental Nutrition Assistance Program (SNAP). The second most common assistance program utilized was free or reduced-price school lunches; 19% of CF parent respondents reported that their children relied on these meals. More than two thirds (68%) of CF adults did not use food assistance resources; over half (56%) of parents of children with CF also noted not leveraging such options. Furthermore, 41% of the adults and 40% of the parents said they would ***not*** access food assistance resources if they met income requirements for these benefits. Additional details of food assistance program utilization rates are summarized in Figure S1.

## Discussion

While the burden of medical costs and self-care regimens has been documented, research is scant on the prevalence of food insecurity and its potential impact on time and financial burdens and, thus, on the health outcomes in this population. Among this group of Idahoans—adults with CF and parents raising children with CF—concerns about food/nutrition supplement costs and accessibility emerged. Compared to the community at large, food insecurity rates were found to be significantly more prevalent among study participants. The percentage of food insecure patients in both study groups (parents of children with CF 33%; adults with CF, 43%) was more than double and nearly triple state levels of food insecurity (15%) (21). Furthermore, the prevalence of food insecurity was higher than reported by an earlier study conducted in other states in the Mountain West region of the United States (26.3%) (23).

The types of foods desired by CF patients implies that food insecurity may inhibit compliance with dietary needs (high-protein/high-fat foods). In addition, the survey found that when the money runs out, patients and families are not able to purchase adequate amounts of food or the medically-necessary nutrition supplements/formulas most CF patients rely on for optimal nutrition. This finding suggests that some patients/parents with limited resources might forgo required medications in order to purchase adequate food. Clinical experience has found that insurance often does not cover these nutrition supplements/formulas (even if prescribed by a provider). Thus, the findings around food insecurity and CF patients emphasize the need to address this gap in insurance coverage. Indeed, optimal dietary intake is necessary for maintaining health in CF patients; therefore, ideally insurers would also cover required food purchases.

The study also explored the correlation between food insecurity and both BMI percentile/BMI and FEV_1_; no significant associations emerged between these health metrics and food security among this CF population. Furthermore, despite the overlap between overweight/obesity and food insecurity among other populations (32, 33), food insecurity was not linked to obesity among study participants.

Research correlates food insecurity with higher utilization of health care. A recent study analyzing food insecurity and health care expenditures in the United States from 2011 to 2013 (*n* = 16,663) found that expenditures for hospitalizations and prescription medications were higher among the food insecure ($493.41 greater per year, *p* = 0.03 and $779.36 greater per year, *p* < 0.0001, respectively) compared to their food secure counterparts (34). A Canadian study (*n* = 67,033, 18–64 years) compared health care costs for inpatient hospital care, emergency department visits, physician services, same-day surgeries, home care services, and prescription medications with severity of food insecurity. Compared to food secure households, health care costs for marginally food insecure households were 16% higher (95% CI, 10–23%); 32% higher for moderate food insecurity (95% CI, 25–39%); 76% higher for severe food insecurity (95% CI, 65–88%) (35). Analysis of the Australian Cystic Fibrosis Data Registry correlated a one point improvement in FEV_1_ percent predicted to a 1.4% decrease in health care costs (17).

The results of this study and previous research demonstrate the value of assessing food security among all aged individuals with CF and their families, and addressing food insecurity as aggressively as possible. While the 6-item survey used for this study could be cumbersome to administer, a two-question tool is available, which has been validated for use with both child and adult populations (36, 37). Some health care organizations have already incorporated this screening into their electronic health records (37, 38). The two food security screening questions ask respondents to indicate “often true, sometimes true, never true, or prefer not to answer” to the following statements:

In the last 12 months, we worried whether (my/our) food would run out before (I/we) got money to buy more.

In the last 12 months, the food that (I/we) bought just didn't last and (I/we) didn't have money to get more (37).

The study has several strengths. The survey tool leveraged the MWCFC-Q validated questions on food insecurity and the participants were both from a defined catchment area and seen at a single center. The study's scope went beyond the traditional evaluation of the impact of CF on children, adding new insights into the quality of life among adults with CF. The small sample size, however, may limit the ability to generalize the findings to CF population at large. In addition, the volunteer participation model risks the introduction of self-selection bias; however, given that about 70% of the St. Luke's Cystic Fibrosis Center of Idaho patients participated in the study the risk for selection bias was diminished. Furthermore, because they were below the age for evaluating lung functioning, FEV_1_ tests were not done on 19 of the 43 children with CF (42.2%); thus, the lack of correlation between food insecurity and lung health may not be generalizable to children age six and under. Lastly, this study did not consider the impact of confounding variables, such as socioeconomic factors often found among food insecure populations (lower income level, less education, single parent households).

## Conclusion

Food insecurity is highly prevalent among Idahoans with CF; rates are significantly above the rate for the general population in the state. Furthermore, food insecurity may impact a patient's ability to adhere to the required dietary modifications and overall treatment protocol for CF, as well as, lessen their chances of an improved quality of life as they age. Though no significant correlations precipitated with regards to food insecurity and lung function or BMI, the importance of providers querying patients/parents about this social determinant of health emerges. A validated, two-question survey tool is available for screening patients and families about food access issues.

One surprising finding in this study was the degree of reluctance among patients/parents to utilize food assistance programs. Further investigation into this issue and how to sensitively encourage patients and families to leverage these programs is needed. Thus, the importance of CF providers (physicians, advanced practice providers, social workers, dietitians, and nurses) being knowledgeable about what food assistance resources are available in their area and coordinating information and referrals for such services surfaces.

Finally, this study, combined with results from McDonald et al. highlights the value of CF advocacy organizations promoting efforts to address and the education around food access concerns (26). For example, additional research designed to enhance the specifics of food insecurity problems among the whole CF population (from pediatric to aging adult), development of innovative assistance programs, improved access and support for current assistance programs, and lobbying for insurance coverage of nutrition supplements/formulas would be highly valuable.

## Data availability statement

The datasets for this manuscript are not publicly available because: St. Luke's Cystic Fibrosis Center of Idaho does not have the capacity to release the data at this time.

## Author contributions

PB contributed to study conception and design, manuscript writing, and final approval of the version being published. DD contributed to study conception and design, data collection, manuscript writing, and final approval of the version being published. RT contributed to data analysis and final approval of the version being published. SS and CW contributed to manuscript writing and final approval of the version being published. ST, BG, and TR contributed to critical manuscript revisions and final approval of the version being published.

### Conflict of interest statement

The authors declare that the research was conducted in the absence of any commercial or financial relationships that could be construed as a potential conflict of interest.

## References

[1] Sawicki GS Sellers DE Robinson WM. High treatment burden in adults with cystic fibrosis: challenges to disease self-management. J. Cystic Fibrosis (2009) 8:91–6. 10.1016/j.jcf.2008.09.007 18952504 PMC2680350

[2] Hurley MN McKeever TM Prayle AP Fogarty AW Smyth AR. Rate of improvement of CF life expectancy exceeds that of general population–Observational death registration study. J Cystic Fibrosis (2014) 13:410–5. 10.1016/j.jcf.2013.12.002 24418187 PMC4074348

[3] Registry CFFP Patient Registry Annual Data Report (2016).

[4] Dodge JA Lewis PA Stanton M Wilsher J. Cystic fibrosis mortality and survival in the UK: 1947–2003. Eur Respir J. (2007) 29:522–6. 10.1183/09031936.00099506 17182652

[5] Borowitz D Baker RD Stallings V. Consensus report on nutrition for pediatric patients with cystic fibrosis. J Pediatric Gastroenterol Nutr. (2002) 35:246–59. 10.1097/00005176-200209000-00004 12352509

[6] Yankaskas JR Marshall BC Sufian B Simon RH Rodman D. Cystic fibrosis adult care: consensus conference report. Chest (2004) 125:1S−39S. 10.1378/chest.125.1_suppl.1S 14734689

[7] Alvarez JA Ziegler TR Millson EC Stecenko AA. Body composition and lung function in cystic fibrosis: association with adiposity and normal weight obesity. Nutrition (2016) 32:447–52. 10.1016/j.nut.2015.10.012 26740256 PMC4769897

[8] Stallings V Stark L Robinson K Feranchak A Quinton H. Evidence-based practice recommendations for nutrition-related management of children and adults with cystic fibrosis and pancreatic insufficiency: results of a systematic review. J Am Diet Assoc. (2008) 108:832–9. 10.1016/j.jada.2008.02.020 18442507

[9] Kalnins D Wilschanski M. Maintenance of nutritional status in patients with cystic fibrosis: new and emerging therapies. Drug Design Dev Ther. (2012):2012–6. 10.2147/DDDT.S9258 22787388 PMC3392141

[10] O'Donohoe R Fullen BM. Adherence of subjects with cystic fibrosis to their home program: a systematic review. Respirat Care (2014) 59:1731–46. 10.4187/respcare.02990 25233386

[11] Stark LJ. Can nutrition counselling be more behavioural? Lessons learned from dietary management of cystic fibrosis. Proc Nutr Soc. (2003) 62:793–9. 10.1079/PNS2003294 15018477

[12] Zemanick E Harris J Conway S Konstan M Marshall B Quittner A. Measuring and improving respiratory outcomes in cystic fibrosis lung disease: opportunities and challenges to therapy. J Cystic Fibrosis (2010) 9:1–16. 10.1016/j.jcf.2009.09.003 19833563 PMC2830746

[13] Sullivan JS Mascarenhas MR. Nutrition: prevention and management of nutritional failure in cystic fibrosis. J Cystic Fibrosis (2017) 16:S87–93. 10.1016/j.jcf.2017.07.010 28986026

[14] Panagopoulou P Fotoulaki M Nikolaou A Nousia-Arvanitakis S. Prevalence of malnutrition and obesity among cystic fibrosis patients. Pediatrics Int. (2013) 56:89–4. 10.1111/ped.12214 24003895

[15] Lai HJ Shoff SM. Classification of malnutrition in cystic fibrosis: implications for evaluating and benchmarking clinical practice performance. Am J Clin Nutr. (2008) 88:161–6. 10.1093/ajcn/88.1.161 18614737 PMC2527817

[16] van Gool K Norman R Delatycki MB Hall J Massie J Understanding the cost of care for cystic fibrosis: an analysis by age and health state. Value Health (2013) 16:345–55. 10.1016/j.jval.2012.12.003 23538187

[17] Gu Y Garcia-Pérez S Massie J van Gool K. Cost of care for cystic fibrosis: an investigation of cost determinants using national registry data. Eur J Health Econom. (2015) 16:709–17. 10.1007/s10198-014-0621-5 25106736

[18] Kettler L Sawyer S Winefield H Greville H. Determinants of adherence in adults with cystic fibrosis. Thorax (2002) 57:459–64. 10.1136/thorax.57.5.459 11978927 PMC1746335

[19] Coleman-Jensen A Nord M Andrews M Carlson S Household Food Security in the United States in 2011. United States Department of Agriculture (2012).

[20] Coleman-Jensen A Rabbitt M Gregory C Singh A Household Food Security in the United States in 2014. United States Department of Agriculture (2015).

[21] Bank IF Food Insecurity in Idaho (2015).

[22] Charkhchi P Fazeli Dehkordy S Carlos RC. Housing and food insecurity, care access, and health status among the chronically ill: an analysis of the behavioral risk factor surveillance system. J Gen Int Med. (2018) 33:644–50. 10.1007/s11606-017-4255-z 29299816 PMC5910337

[23] McDonald C Christensen N Lingard C Peet K Walker S Nutrition knowledge and confidence levels of parents of children with cystic fibrosis. ICAN (2009) 1:325–31. 10.1177/1941406409355192

[24] Steinkamp G Wiedemann B. Relationship between nutritional status and lung function in cystic fibrosis: cross sectional and longitudinal analyses from the German CF quality assurance (CFQA) project. Thorax (2002) 57:596–601. 10.1136/thorax.57.7.596 12096202 PMC1746376

[25] Goodin B(2005). Nutrition issues in cystic fibrosis. Pract Gastroenterol, 27:76–94. Available online at: https://www.practicalgastro.com/pdf/GoodinArticle_May05.pdf

[26] Cruz I Marciel KK Quittner AL Schechter MS. Anxiety and depression in cystic fibrosis. Semin Respir Crit Care Med. (2009) 30:569–78. 10.1055/s-0029-1238915 19760544

[27] Harris PA Taylor R Thielke R Payne J Gonzalez N Conde JG. Research electronic data capture (REDCap)–a metadata-driven methodology and workflow process for providing translational research informatics support. J Biomed Inform. (2009) 42:377–81. 10.1016/j.jbi.2008.08.010 18929686 PMC2700030

[28] Bickel G Nord M Price C Hamilton W Cook J Measuring food security in the United states - guide to measuring household food security. In: Drive PC Alexandria V, editors. Office of Analysis N, and Evaluation, Food and Nutrition Service USDA, Alexandria, VA (2000). p. 305–2133.

[29] Stanojevic S Wade A Stocks J Hankinson J Coates AL Pan H. Reference ranges for spirometry across all ages: a new approach. Am J Res Crit Care Med. (2008) 177:253–60. 10.1164/rccm.200708-1248OC 18006882 PMC2643211

[30] Piccioni P Borraccino A Forneris M Migliore E Carena C Bignamini E. Reference values for forced expiratory volumes and pulmonary flows in 3–6 year children: a cross-sectional study. Respir Res. (2007) 8:14. 10.1186/1465-9921-8-14 17316433 PMC1810252

[31] Centers for Disease Control and Prevention. Body Mass Index. (2015). 29540783

[32] Kaisere ML Cafer A Understanding high incidence of severe obesity and very low food security in food pantry clients: implication for social work. Social Work Public Health (2018) 33:125–39. 10.1080/19371918.2017.1415181 29297775

[33] Food Research and Action Center Understanding the Connections: Food Insecurity and Obesity (2015).

[34] Berkowitz S Basu S Meigs J Seligman H. Food insecurity and health care expenditures in the United States, 2011–2013. Health Serv Res. (2017) 53:1600–20. 10.1111/1475-6773.12730 28608473 PMC5980147

[35] Tarasuk V Cheng J de Oliveira C Dachner N Gunderson C Kurdyak P. Association between household food insecurity and annual health care costs. Can Med Assoc J. (2015 187:E429–36. 10.1503/cmaj.150234 26261199 PMC4592315

[36] Swindle TM Whiteside-Mansell L McKelvey L Food insecurity: validation of the two-item screen using convergent risks. J Child Family Stud. (2013) 22:932–41. 10.1007/s10826-012-9652-7

[37] Gundersen C Engelhard EE Crumbaugh AS Seligman HK. Brief assessment of food insecurity identifies high-risk US adults. Public Health Nutr. (2017) 20:1367–71. 10.1017/S1368980017000180 28215190 PMC10261547

[38] Council on Community Pediatrics CoN Promoting food security for all children. Pediatrics (2015) 136:1431–8. 10.1542/peds.2015-3301 26498462

