# Supplementary material for: Evaluation of Food Insecurity in Adults and Children With Cystic Fibrosis: Community Case Study
Source: Front Public Health. 2018 Nov 26;6:348. doi: 10.3389/fpubh.2018.00348 (PMC6275191; doi:10.3389/fpubh.2018.00348)

Supplementary Material

Evaluation of Food Insecurity in Adults and Children with Cystic Fibrosis: Community Case Study

Perry S. Brown[1*], Dixie Durham[1*], Rick Tivis[2], Shannon Stamper[1], Cleary Waldren[3], Sarah Toevs[4,5], Barbara Gordon[2], Tiffany Robb[4]

[1]St. Luke's Cystic Fibrosis Center of Idaho, Boise, Idaho, United States

[2]Sam and Aline Skaggs Health Science Center, Idaho State University, Meridian, Idaho, United States

[3]St. Luke's Health System, Boise, Idaho, United States

[4]Center for the Study of Aging, Boise State University, Boise, Idaho, United States

[5]Community and Environmental Health, Boise State University, Boise, Idaho, United States

*** Correspondence:**
Dr. Perry Brown
perry.brown@fmridaho.org

*** Correspondence:**
Dixie Durham
durhamd@slhs.org

Supplementary Figure

Figure 1. Food Assistance Programs Utilized by CF Patients

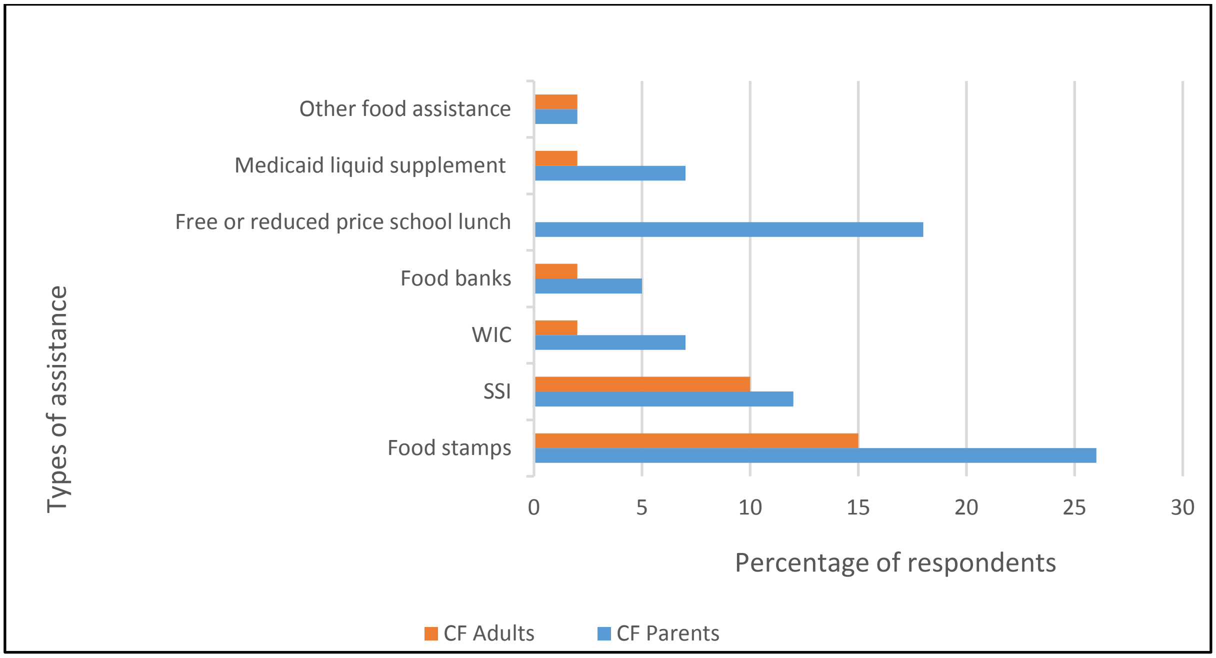

Supplement: Supplementary file 1 [file Data_Sheet_1.PDF]
